# Siloxanes—Versatile Materials for Surface Functionalisation and Graft Copolymers

**DOI:** 10.3390/ijms21176387

**Published:** 2020-09-02

**Authors:** Karolina Glosz, Agnieszka Stolarczyk, Tomasz Jarosz

**Affiliations:** Department of Physical Chemistry and Technology of Polymers, Silesian University of Technology, 9 Strzody Street, 44-100 Gliwice, Poland; Karolina.Glosz@polsl.pl (K.G.); Agnieszka.Stolarczyk@polsl.pl (A.S.)

**Keywords:** siloxane, surface modification, functionalisation, polysiloxane, graft copolymer

## Abstract

Siloxanes are adaptable species that have found extensive applications as versatile materials for functionalising various surfaces and as building blocks for polymers and hybrid organic-inorganic systems. The primary goal of this review is to report on and briefly explain the most relevant recent developments related to siloxanes and their applications, particularly regarding surface modification and the synthesis of graft copolymers bearing siloxane or polysiloxane segments. The key strategies for both functionalisation and synthesis of siloxane-bearing polymers are highlighted, and the various trends in the development of siloxane-based materials and the intended directions of their applications are explored.

## 1. Introduction

The Si-O bond is a highly versatile chemical linkage that can be found in a great variety of materials and molecules, linking together inorganic [1,2,3] and organic species [4,5,6], as well as being a building block for polymers [7,8,9] and sophisticated 3D oligomers [10,11]. In the role of linkage, the siloxane bond is robust, chemically resistant to an array of environments and easily established, e.g., via the reaction of silanes with organic or inorganic hydroxyl groups [6,12,13]. Conversely, systems containing repeating siloxane bonds—polysiloxanes—tend to exhibit good mechanical properties (ranging from elastomeric to more rigid, dependent on the molecular weight of the polymers, introduced substituents and the use of cross-linking reactions), medium-high solubility in common organic solvents, self-assembling and film-forming properties, as well as low dielectric constants and, frequently, biocompatibility [14]. 

Consequently, siloxanes have found application for the modification of various surfaces [2,15,16], bestowing upon them a variety of properties, such as increased hydrophobic nature [17]. Conversely, oligo- and polysiloxanes are more commonly employed as primary or auxiliary materials in optoelectronics [18], for biomedical purposes [19] and as protective coatings [20].

In recent years, a multitude of works dedicated to the synthesis and application of both siloxanes and polysiloxanes have been published. Despite this, only a fraction of the reports shows significant progress, whether in terms of scientific novelty or material properties. Consequently, this review is dedicated to the most recent highlights (published since the beginning of 2017) in the field of siloxane-based materials, with mentions of individual works being categorised by the type and nature of the siloxane system being reported.

## 2. Synthetic Routs for Producing Siloxanes

**Figure 1:**
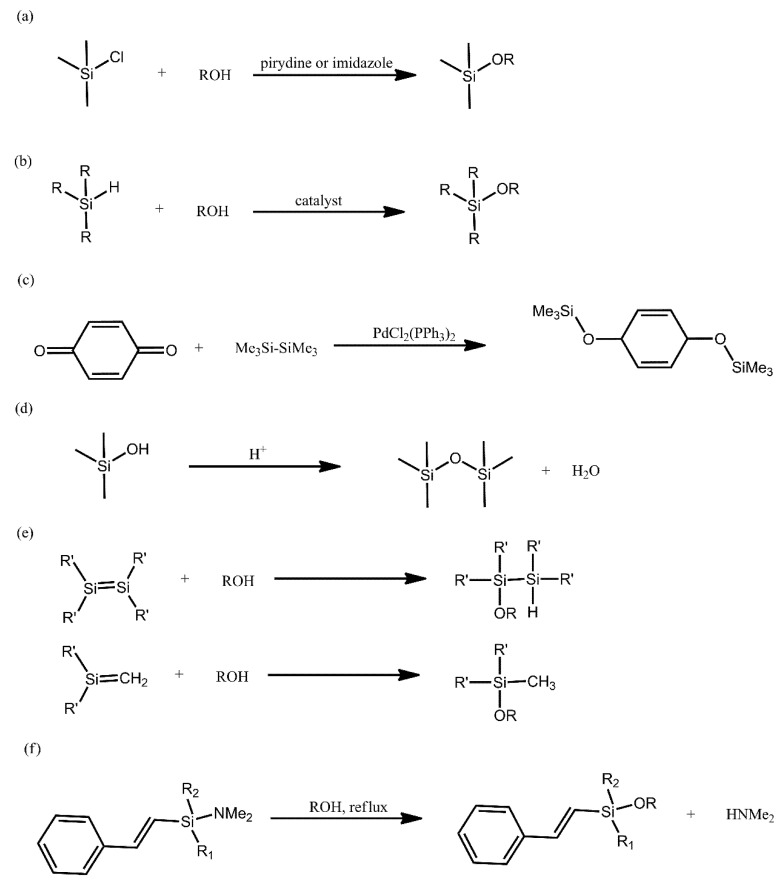
Overview of the most common synthetic methods for producing siloxanes.

The Si-O bond is the linkage that is most commonly encountered in nature between silicon and a heteroatom. This is due to the strong nature of this bond, resulting in, e.g., Si-C bonds being replaced by Si-O bonds and, eventually, the formation of oligomeric siloxane structures. Such structures show higher flexibility and are more inert chemically in comparison with analogous systems, based on chains constituted by C-C bonds. These properties, among others, are the reason for the significant research interest in polysiloxanes and other organosilicon compounds. Below, we provide a brief overview of the most common synthetic methods for producing such compounds (Figure 1).

Alkoxysilanes are widely used in chemistry, since they can act as linkers between both organic and inorganic species, forming hybrid compounds.

The most common method for producing alkoxysilanes is the condensation of chlorosilanes with alcohols, being typically performed in alkaline environments and in the presence of such bases as pyridine, imidazole and tertiary amines (Figure 1a). A significant drawback of this reaction is the need to employ acid scavenger chemicals, in order to remove the evolving acid from the reaction environment. This is due to the fact that the acid can promote Si-O bond cleavage [21].

Alcoholysis of hydrosilanes is a promising alternative to the above method. The advantage of this reaction is that hydrogen is its only by-product (Figure 1b). The key disadvantage of this approach is the need to employ a catalyst under ambient conditions, because alcohols are not sufficiently nucleophilic. Consequently, research on finding suitable catalysts for this reaction has been on-going for many years, yielding a variety of results, each with their own drawbacks and advantages. This has taken us from catalysts that required elevated temperature, through catalysts that promoted the formation of harmful by-products and onwards. One of the most recent works devoted to that aspect deals with a system composed of 2,2,2-trifluoroacetophenone in the presence of hydrogen peroxide, which was used to obtain silanols with high yields [22].

Alkoxysilanes can also be obtained with the use of dihydroxyaromatic compounds, via the reducing disililation of quinones, with Pd- and Rh-based catalysts being typically used in this reaction (Figure 1c). Many recent works on the synthesis of alkoxysilane oligomers have turned to using this method.

In order to induce the formation of a Si-O bond, acid-catalysed condensation of silanols can also be conducted (Figure 1d). This reaction is frequently employed for obtaining linear siloxanes, silsesquioxanes, silica networks and glasses. The use of strong acids, however, leads to the equilibrium of the reaction being rapidly achieved. 

Unsaturated silicon compounds are highly reactive and can yield alkoxysilanes when treated with alcohols (Figure 1e). Due the lack of a catalyst, the nucleophilic addition of water and alcohols to disilenes requires elevated temperatures and takes place at a significantly slower rate than the nucleophilic addition for silenes. 

Due to the unstable nature of Si-N bonds, they can be readily converted into Si-O bonds and such a reaction is employed, e.g., for producing sillyl ethers attached to phenol moieties (Figure 1f) [21].

## 3. Functionalisation of Surfaces

### 3.1. Functionalisation with Polysiloxane Grafts

Mesoporous carbon, which has been treated to possess carboxyl groups on its surface, is reported to be readily functionalised with aminopropyl-terminated poly (dimethylsiloxane) (PDMS) [23] via the formation of carboxylates and their conversion to amides. The aim of such a functionalisation was to improve both the electrical properties and cesium ion sorption capacity of the carbon. Functionalisation appears to take place uniformly on the surface of the carbon and largely preserves the morphology of the treated carbon. In terms of electrical properties, functionalisation leads to a 3-5-fold increase in conductivity, depending on the method used for treating carbon (0.22 mS/cm for the untreated carbon; 0.63 and 1.1 mS/cm for functionalized carbon treated by peroxide and plasma respectively) to produce carboxyl groups on its surface. In terms of cesium uptake, the treated carbon (both by peroxide and by plasma) shows a capacity of 0.1 mg cesium cations per gram of carbon, whereas the functionalized carbon shows capacities of 48.1 and 25.9 mg/g for peroxide and plasma-treated carbon respectively, showing a many fold improvement, making the material a potential solution for cesium removal applications.

An interesting approach to functionalising silica surfaces with polysiloxane grafts is to employ a siloxane bond-breaking reagent (dimethyl carbonate or diethyl carbonate) on a mixture of a poly (organosiloxane) and nanosilica particles [24]. These species were then investigated in detail by ^1^H, ^13^C and ^29^Si NMR spectroscopy, with all the observed signals being assigned to particular chemical species grafted onto the surface of the silica nanoparticles.

A continuation of this work, limited to the use of dimethyl carbonate and PDMS to functionalise silica nanoparticles, was carried out, focusing on the adsorption of PDMS oil on the modified nanoparticles and on the molecular dynamics of the obtained systems [25]. PDMS with various molecular weights were used (4–40·kg/mol), yielding a total coverage of the silica grain surfaces in the case of low-MW PDMS and a lower coverage (≈60%) for high-MW (molecular weight) PDMS. Although there is no mention of the amount or mass of grafted PDMS or the siloxane bond-breaking catalyst, such a trend would be easily predicted, assuming constant amounts of PDMS and dimethyl carbonate are used, regardless of the MW of the employed PDMS. This is because for high-MW PDMS the chain count in a unit mass of the polymer will be significantly lower than for low-MW PDMS. Even though dimethyl carbonate, which is consumed upon reaction with a siloxane bond, would increase the chain counts, the absolute chain count increases would be the same, regardless of the MW of PDMS.

**Figure 2:**
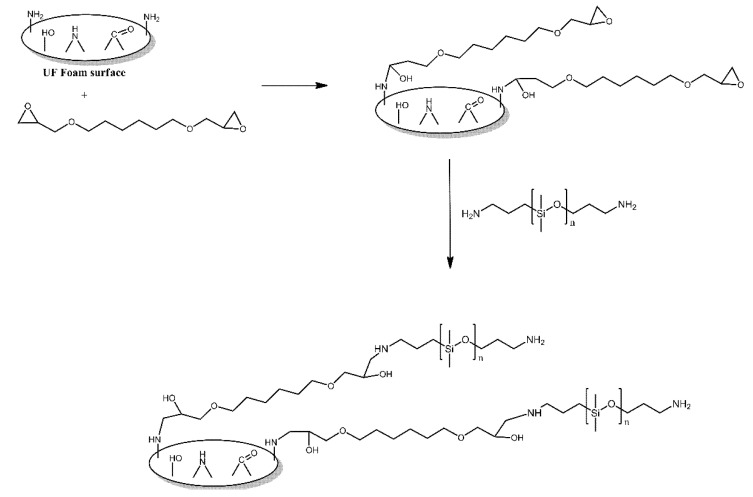
Schematic illustration of urea-formaldehyde foam surface grafted with aminopropyl-terminated PDMS via the bridging effect of 1, 6-hexanediol diglycidyl ether. Based on Ref. [5].

The surface of urea-formaldehyde foams was functionalised with polysiloxane grafts in order to make it more hydrophobic [5]. First, the foams were treated with 1,6-hexanediol diglycidyl ether, utilising the reaction of its epoxy functionalities with the amine functionalities present on the surface of those foams. Subsequently, the foams were treated with aminopropyl-terminated PDMS, which reacted with the remaining epoxy functionalities of the grafted 1,6-hexanediol diglycidyl ether molecules (Figure 2). The modified foams were found to indeed be more hydrophobic than the unmodified foams, with their contact angle values for water being reported as 143.4° and 123.6°, respectively. 

**Figure 3:**
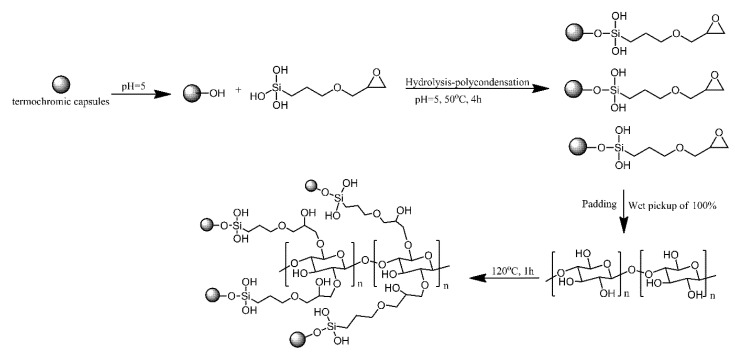
Schematic of thermochromic functionalized cotton fabric with epoxy modified thermochromic capsule. Based on Ref. [4].

Zang proposed a method for obtaining reversible, temperature-induced colour changing cotton fabric by grafting to cotton fabric via reacting with epoxy modified thermochromic capsule. Epoxy modified thermochromic capsule was prepared by the hydrolysis-polycondensation of siloxane groups of 3-glycidoxypropyltrimethoxysilane with the hydroxy groups of the capsules. The epoxy groups on the surface of thermochromic capsules reacted with the hydroxy groups of cotton fabric and formed covalent bonds (Figure 3) [4]. The modified cotton fabrics changed reversibly from blue to white, the same as the capsules that were not bonded to the fabric. The covalent bonding between the fabric and the capsules resulted in excellent washing and rubbing resistance.

### 3.2. Functionalisation with Siloxanes

An interesting approach to the functionalisation of alloys is reported for a HfNbTaTiZr alloy [12]. The surface of this alloy was first oxidised, so as to functionalise it with hydroxyl groups, followed by treatment with oligo (ethylene glycol) or alkylamino-equipped trimethoxysilanes in ethanol. By reacting with the surface hydroxyl groups, the silanes were transformed into siloxanes and bound to the surface of the alloy. This surface modification method was found to prevent metal release from the surface of the alloy, when immersed in phosphate-buffered saline for an extended period, a desirable feature for potential bioimplant applications.

Epoxy-equipped polyhedral oligomeric silsesquioxanes (POSS) were grafted onto the surface of carbon fibres as part of the procedure for producing carbon fibre-reinforced epoxy composites [16] for potential applications in low earth orbit environments. The carbon fibres were first subjected to several reactions, in order to functionalise them with amino groups. These groups were then reacted with the epoxy functionalities of the POSS molecules. The modified carbon fibres were then treated with an epoxy resin and curing agent, in order to produce the final composites. The resultant composite exhibited improved adhesion between the epoxy phase and the carbon fibre reinforcement, possibly due to curing taking place between the POSS epoxy functionalities and the epoxy resin, as well as improved resistance to atomic oxygen erosion and increased interlaminar shear strength. The composites were prepared by reacting amine groups on the pre-treated carbon fibre surface with the POSS to form a continuous uniform layer of siloxane oligomers. X-Ray photoelectron spectroscopy, scanning electron microscopy and infrared (IR) spectroscopy demonstrated that POSS was successfully grafted onto the carbon fibres surface.

An interesting method for obtaining dendrite-free lithium batteries was proposed by Meng et al. [3]. Utilizing the ability to attach siloxanes to hydroxyl groups on the lithium metal surface with the formation of a siloxane metal bond. Lithium as the active metal has hydroxyl groups on its surface, so the authors apparently used them to react with a liquid, methoxy-terminated PDMS, producing a layer on the lithium metal surface, in order to achieve deposition of uniform lithium layers for batteries.

### 3.3. Functionalisation with Other Moieties via Siloxane Bonds

**Figure 4:**
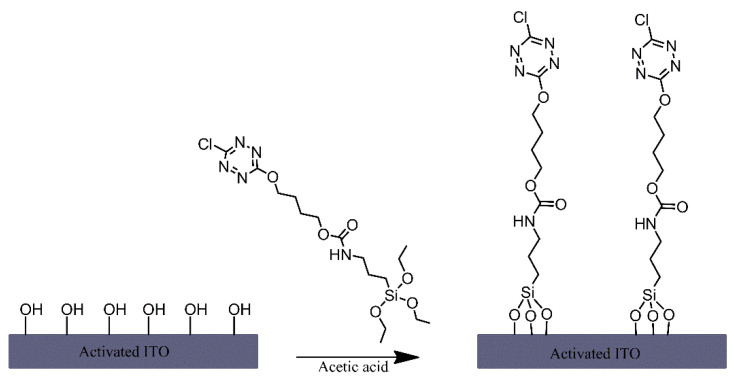
Formation of tetrazine terminated alkyl chain monolayers on indium-tin oxide (ITO). Based on Ref. [1].

A triethoxysilane-functionalised tetrazine derivative was grafted onto the surface of indium-tin oxide (ITO) electrodes via immersing the electrodes in a solution of this derivative that also contained acetic acid (Figure 4) [1]. Although no mechanism is mentioned, the authors have likely relied on the presence of hydroxyl groups on the ITO surface and their reaction with the silane-functionalised tetrazine to form siloxane bonds for modifying the electrodes. The tetrazine-modified electrodes were found to exhibit notable fluorescence, which was dependent on the oxidation state of the tetrazine moiety, making it an interesting material for potential sensing or display applications.

**Figure 5:**
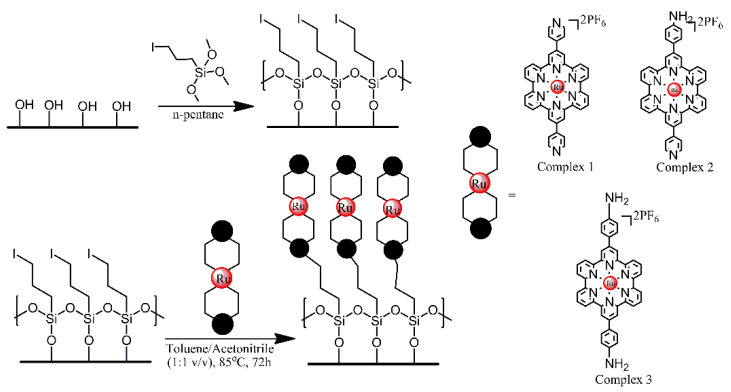
Schematic Representation of the Two-Step Fabrication Process for the Covalent Assembly of Functional Ru(II) Complexes 1−3 on Freshly Activated, Hydroxylated SiOx-Based Substrates (Si/Glass/ITO), Resulting in the Formation of metalloligand monolayers. Based on Ref. [13].

Glass, ITO and Si (100) wafer surfaces were modified with Ru^2+^ terpyridyl complexes, in order to produce materials for Hg^2+^ sensing [13]. First, the substrate surfaces were treated with an iodo-functionalised trimethoxysilane, utilising its reaction with the hydroxyl groups present on those surfaces. Subsequently, the iodo-functionalised surfaces were treated with one of the three terpyridyl derivatives, via a S_N_2 coupling reaction between the iodo-functionalised alkyl chains and pendant pyridyl groups or primary amino group of the complexes (Figure 5). The optical and electrochemical properties of the respective functional monolayers were studied in detail and their potential for detecting ppm levels of highly toxic Hg^2+^ ions was tested.

**Figure 6:**
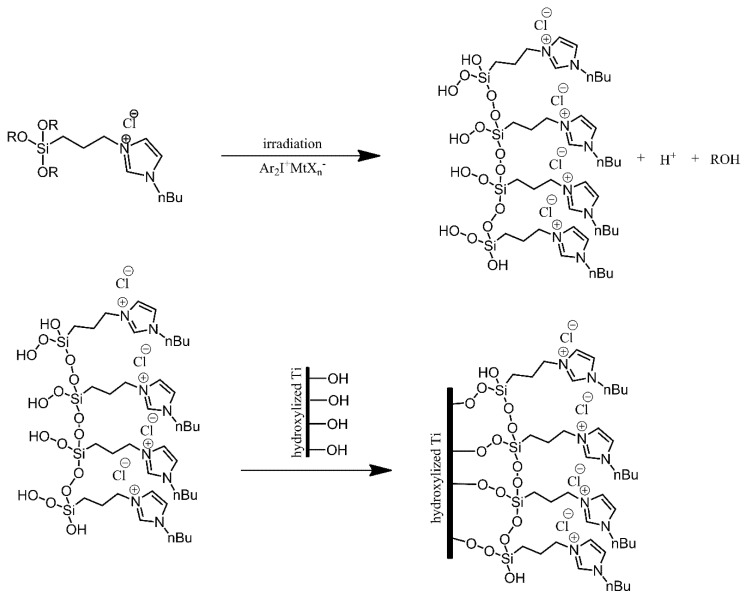
Photografting of the imidazolium-derived siloxane on a titanium plate. Based on Ref. [2].

Titanium substrates were modified using imidazolium-terminated trialkoxysilanes, in order to produce surfaces resistant to bacterial colonization [2]. The titanium substrates were first treated, cleaned with ethanol and toluene, and then oxidised under acidic conditions. Next, instead of directly reacting the alkoxysilanes with hydroxyl groups present on the surface of the substrates, the alkoxysilanes were irradiated, resulting in cross-linking between neighbouring silane molecules and introduction of hydroperoxide substituents to the silicon atoms. These hydroperoxide functionalities were then used to anchor the silanes onto the substrates via the hydroxyl groups on their surface (Figure 6). The modification of the substrates resulted in a significant improvement in terms of the resistance of the surfaces to bacterial colonisation by *E. coli* and *S. aureus*. Unfortunately, this resistance was found to be short-term, as it was lost after approximately 24 h.

**Figure 7:**
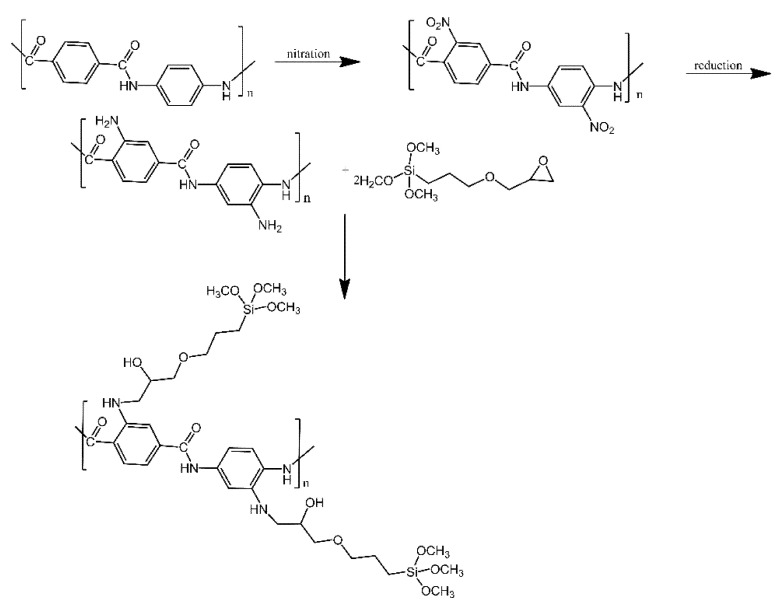
Mechanism for preparation of g-(amino groups aramid fibre). Based on Ref. [15].

Silica aerogel surfaces were functionalised with modified aramid fibres, in order to produce materials with lower thermal conductivity [15]. Aramid fibres were first nitrated, introducing nitro groups into their aromatic rings. These nitro groups were then reduced to amino groups, which in turn were treated with epoxy-functionalised silanes. Similarly to other works mentioned here, the silanes were used to couple with the hydroxyl groups present on the silica aerogel surfaces and anchor the grafts to them (Figure 7). Only a small decrease in thermal conductivity was observed (thermal conductivity coefficient of 0.032 W/m·K for the silica-modified fibres, in comparison for 0.042 W/m·K for the non-modified aramid fibres).

**Figure 8:**
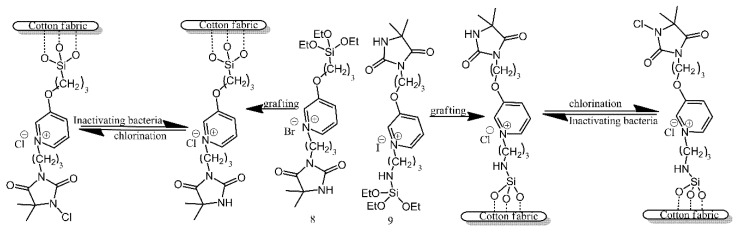
Schematic illustration of cotton fabric grafting and chlorination. Based on Ref. [6].

Two types of pyridinium *N*-chloramine precursors were anchored to the surface of cotton utilising the reaction of their alkoxysilane functionalities with the hydroxyl groups present on the surface of cotton (Figure 8) [6]. Following grafting, the precursors needed to be chlorinated with bleach, in order to temporarily bestow upon them antibacterial properties. The treated and activated surfaces were found to be effective against *E. coli* and *S. aureus*, although there is no mention of the duration for which these properties persisted.

**Figure 9:**
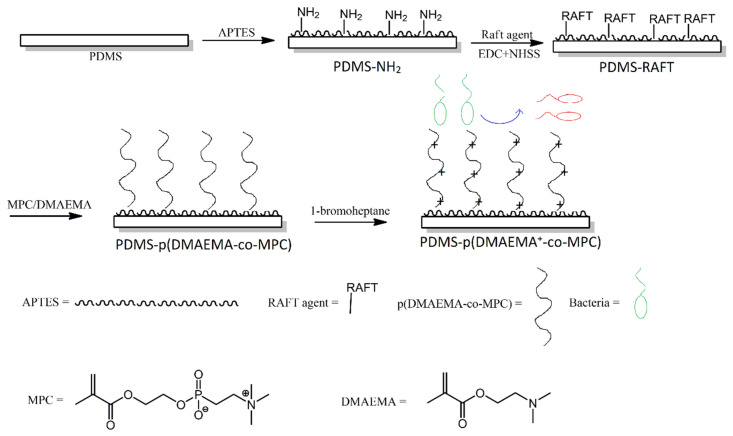
Schematic illustration of the preparation of p(DMAEMA^+^-co-MPc) copolymer brushes on poly (dimethylsiloxane) (PDMS) with nonfouling and bactericidal properties. Abbreviations: p (DMAEMA^+^-co-MPc) = (2-(dimethylamino)-ethyl methacrylate-co-2-methacryloyloxyethyl phosphorylcholine; APTES = (3-aminopropyl)triethoxysilane; EDC = N-(3-dimethylaminopropyl)-N’-ethylcarbodiimide hydrochloride; NHSS = N-hydroxysulfosuccinimide sodium salt. Based on Ref. [26].

An interesting approach to producing non-fouling and antibacterial multifunctional surfaces was reported by Wang et al. First, PDMS was treated with an amino-functionalised silane, in order to produce amine groups on the surface of PDMS. Next, those amino groups were utilised in a reversible addition-fragmentation chain transfer (RAFT)-type polymerisation reaction, to produce methacrylate copolymer chains that contained phosphorylcholine pendant groups (Figure 9). Lastly, the methacrylate copolymer chains were treated with bromoheptane, in order to convert their amine functionalities into quaternary ammonium moieties. The modified surfaces showed efficiency in reducing bovine serum albumin adsorption, inhibiting bacteria adhesion and biofilm formation, as well as bactericidal properties towards *S. aureus* [26].

## 4. Graft Copolymers Bearing Polysiloxane Units

### 4.1. Polysiloxanes with Non-Polymer Grafts

Polysiloxanes have been equipped with benzene-1,3,5-tricarboxamide (BTA) grafts, via the addition of an alkene-functionalised BTA to poly (dimethylsiloxane-co-methylhydrosiloxane) (PDMS-co-MHS) [9]. The BTA functionalities were introduced as folding promoters, in order to achieve a supramolecuar organisation of the individual polymer chains. PDMS-co-MHS of different molecular weight (MW) and MHS content was employed to achieve different densities of BTA grafts, with the polymers transitioning from a flexible solid, in the case of a low BTA graft density (high-MW and low-MHS content in the PDMS-co-MHS polymer), to a brittle solid, at a higher BTA graft density (low-MW and high-MHS content). The BTA-decorated polymers were found to form helical aggregates, with the degree of aggregation being dependent on the density of the BTA grafts in the polymer.

Polysiloxanes and polysilsesquioxanes bearing vinyl groups were grafted with N-acetylcysteine molecules via a thiol-alkene addition [27]. The functionalised materials were then used to produce thin films and compared with N-acetylcysteine in terms of their efficacy against common bacteria and found to exhibit good performance in eradicating biofilms.

**Figure 10:**
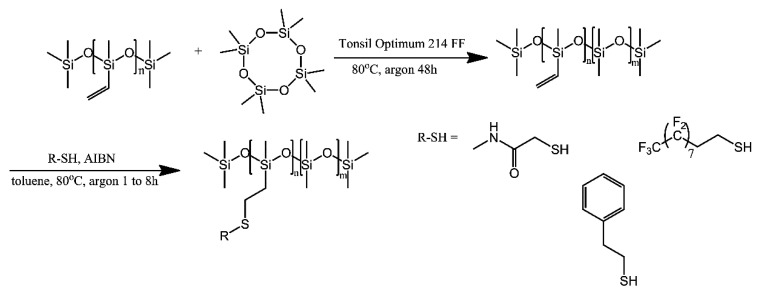
General scheme for the synthesis of functionalised polysiloxanes. Based on Ref. [27].

Vinyl-equipped polysiloxanes were equipped with various small-molecular grafts via the thiol-ene coupling reaction [28]. First, commercial vinyl-bearing oligosiloxanes were coupled with octamethyltetrasiloxane to produce polysiloxanes with the desired vinyl group content. These vinyl-equipped polysiloxanes were then grafted with various small molecules via treatment with various thiols or their mixtures, as part of the thiol-ene coupling reaction (Figure 10). The resultant modified polysiloxanes were then used to coat gold nanoparticles, inducing shifts in the surface plasmon resonance bands, based on the polarity and, possibly, refractive index of each modified polysiloxane. Since some of the polymers were found to be soluble in N,N-dimethylformamide or dimethyl sulfoxide, the authors suggest their potential use for preparing carbohydrate grafted polysiloxanes (“glycosilicones”).

**Figure 11:**
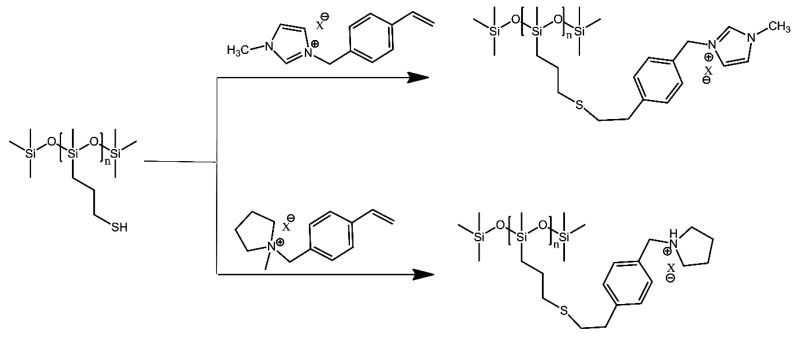
Synthetic routes to produce well-defined grafted PILs. Based on Ref. [29].

Thiol-functionalised polysiloxanes were equipped with imidazolium- and pyrrolidinium-derived grafts using a “grafting to” approach [29]. The authors employed the thiol-ene coupling photo-polymerisation, which is a commonly used high-yield method that can be controlled to a significant extent (Figure 11). In this case, the resultant graft copolymers were polymeric ionic liquids, intended as future high-performance polyelectrolyte materials.

**Figure 12:**
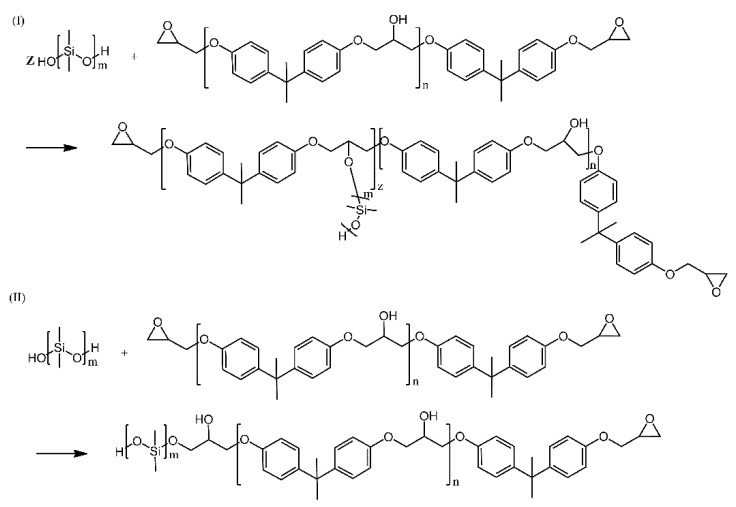
(**I**): dehydration, (**II**): etherification of hydroxyl groups between silicon oil and epoxy resin. Based on Ref. [30].

Hydroxyl-terminated PDMS was also used alongside a bisphenol-A-type epoxy resin and magnesium powders to produce Mg-filled coatings, designed as materials for cathodic protection of aluminium alloy surfaces. The PDMS was included in the composition in order to improve the flexibility and impact resistance of the resultant coatings, as well as to improve compatibility of the polymer phase with magnesium. Interestingly, the reaction system allowed for both dehydration and etherification (Figure 12) to take place, leading to graft and (multi) block copolymers, respectively, with the latter being the prevalent reaction, based on IR spectroscopic investigations [30].

A new graft copolymer was produced via the hydrosilylation reaction of a Si-H functionalised polysiloxane and allyl polyglycidyl ether. The resultant graft copolymer was found to be soluble in solvents of an average dielectric constant value (e.g., isopropanol and dichloromethane), while being insoluble in both high- and low-dielectric constant solvents [31].

### 4.2. Polysiloxane with Polymer Grafts

In a continuation of their earlier work on PDMS-graft polymer [32], the authors used an azide-alkyne click chemistry reaction, conducted between an alkyne-terminated PDMS and azidated poly (arylene ether sulfone), to equip the latter polymer with PDMS grafts [33]. The copolymer was used as a lubricant and found to exhibit good optical transparency and dewetting properties against solvents improved in comparison to the parent homopolymers.

A worthwhile approach to producing polymer composites is gamma ray irradiation [34], even though the chemical structure of the resulting substance will be poorly defined, as grafting, cross-linking and polymer chain scission can all take place during the process. A good example of this approach is a report dedicated to producing composites containing PDMS and either linear low-density polyethylene or isotactic polypropylene. Although the presence of both PDMS and polyolefin segments was confirmed in the composite and elution with solvents did not allow removing either of the segments from the composite, the authors were unable to elucidate its chemical structure. In terms of performance of the composites, the results were in line with expectations, i.e., blending with PDMS resulted in deterioration of viscoelastic properties, but significantly decreased abrasive wear of the materials.

**Figure 13:**
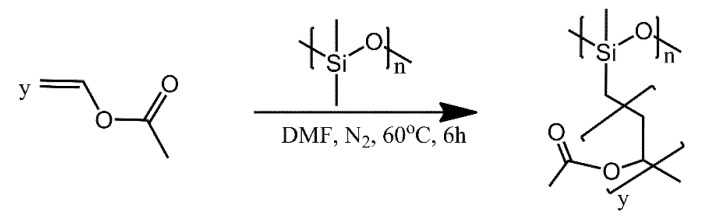
Synthesis of the graft copolymer PDMS-g-PVAc by free radical polymerization. Based on Ref. [35].

A polysiloxane grafted with poly (vinyl acetate) (PVAc) was synthesised (Figure 13) via the free radical polymerisation of vinyl acetate and a low molecular weight PDMS [35]. The aim behind the synthesis of this copolymer was to develop a material alternative to fluoropolymers for application as easy-cleaning coatings. In their work, the authors obtained a series of materials with different co-monomer contents. The results of experimental work were in good accordance with density functional theory (DFT) simulations. PDMS-g-PVAc with 20% PVAc displayed similar performance as poly(tetrafluoroethylene) (PTFE) coating with analogous WCA value (99° vs. 100°), slightly improved transparency (95% versus 94%), and comparable surface energy (21.77 versus 22.08 mJ/m^2^).

**Figure 14:**
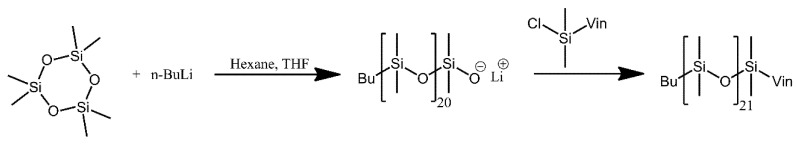
Synthesis of the PDMS arm. Vin stands for a vinyl group. Based on Ref. [36].

A series of star-shaped PDMS derivatives was produced via the “grafting onto” method, utilising various siloxane-derived cores (Figure 14) [36]. Vinyl-terminated PDMS arms with a narrow molecular mass distribution [polydispersity index (PDI) = 1.13] were synthesised via a living anionic polymerization.

The star-shaped polymers were produced by hydrosilylation of PDMS with a vinyl end group and cyclic cores, which contained Si-H groups, in the presence of Karstedt’s catalyst.

**Figure 15:**
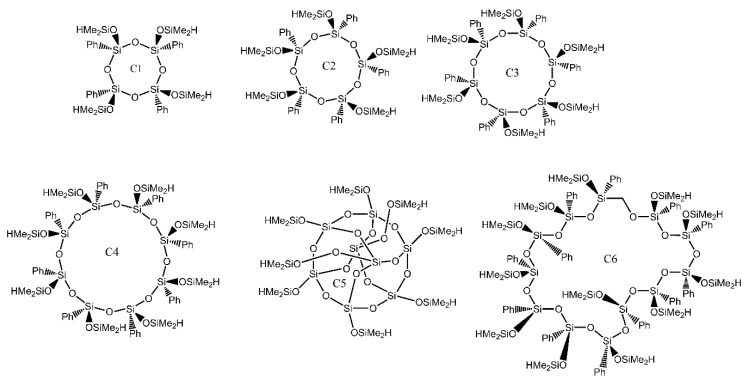
Synthesis of functional cores for further synthesis of star shaped PDMS. Based on Ref. [36].

Functional cyclic phenylsilsesquioxanes were used as the cores. These compounds were obtained from polyhedral metallasiloxanes containing nickel, copper, dysprosium, or sodium ions, and having a well-defined spatial structure. Depending on the metal type, such molecules contain one or two stereoregular cyclosiloxanolate fragments attached to the metal ion matrix20 (Figure 15)

### 4.3. Polymers with Polysiloxane Grafts

Cellulose was modified, via its hydroxyl groups, with PDMS grafts using a ring-opening polymerisation protocol [37]. Copolymers with different PDMS graft lengths were investigated and for sufficiently long grafts, the Johnson–Kendall–Roberts work of adhesion and the adhesion energy at the critical energy release rate were increased by 190% in comparison with unmodified cellulose, making the copolymer a potential material for studying interactions between different polymer surfaces.

A similar modification of cellulose was conducted via plasma polymerisation of hexamethyldisiloxane [38]. Although it is unclear whether a true graft copolymer structure was achieved, the siloxane-treated cellulose was found to indeed exhibit superhydrophobic properties, even reaching water contact angle values of more than 160⁰, unlike cellulose. It is worth mentioning that the hydrophobicity was maintained both after medium-term storage and laundering of the treated material.

A poly (glycidyl methacrylate) grafted with PDMS was produced via radical polymerisation of glycidyl methacrylate and a methacrylate-terminated PDMS [32]. The graft copolymer was then used as one of several components of a lubricating coating formulation.

An acryl polymer with PDMS grafts was prepared by polymerising N-isopropylacrylamide with a PDMS macromonomer, which was on one end terminated with a methacrylate group [39]. It is worth mentioning that radical polymerisation of the two monomers was initiated using a complex of triethylborane with an aliphatic amine. In this complex, the nitrogen-boron coordination has a stabilising role until the bond is broken, e.g., by protonating the amine. Subsequently, triethylborane reacts with oxygen present in the reaction environment, producing radicals and initiating polymerisation. The reactivity coefficients for the two monomers were investigated in detail. Due to the lack of miscibility of the polyacrylate chains with the PDMS grafts, the copolymers were found to undergo de-mixing above a certain temperature, dependent on the PDMS content of the copolymer.

PDMS bridges were also incorporated as modifications of bio-originating molecules [40], via the reaction of an epoxy-terminated PDMS with the amine groups present in collagen hydrolysate macromolecules, yielding a class of biodegradable surfactants. In terms of their properties, the surfactants produced using low-MW collagen hydrolysate achieved a higher PDMS grafting degree and exhibited more favourable surface activity, foaming ability and emulsifying capacity than the surfactants produced using higher-MW collagen hydrolysate, although all of the systems were considered to exhibit favourable properties.

**Figure 16:**
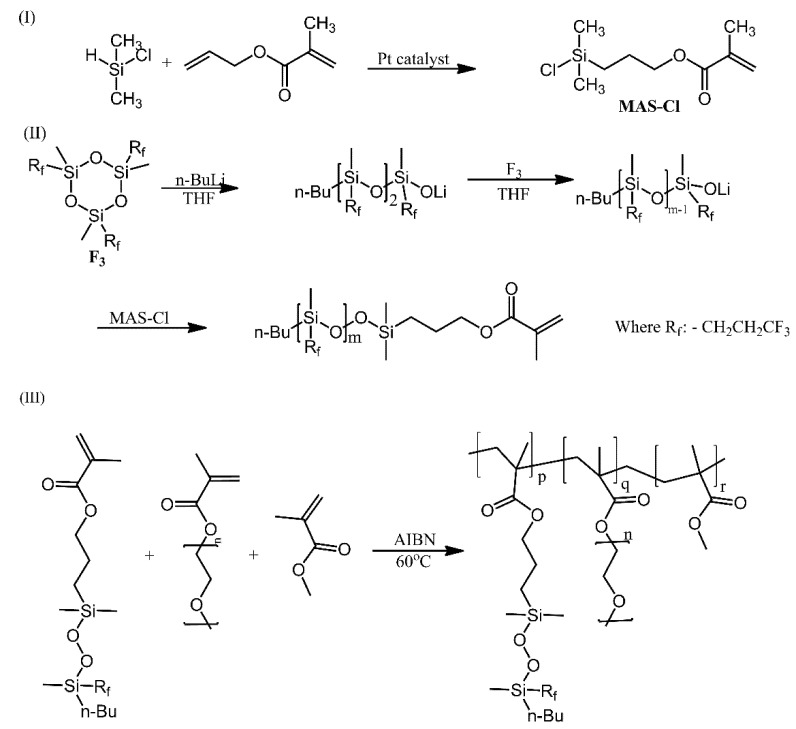
Synthesis of PMTFPS-MA macoromonomers and amphiphilic PMMA-g (PEG, PMTPPS) graft copolymers. AIBN: Azobisisobutyronitrile. Based on Ref. [8].

Poly (methyl methacrylate) equipped with polysiloxane and poly (ethylene glycol) (PEG) grafts was developed as a new amphiphilic material [8]. Interestingly, a “grafting through” approach was employed, via the radical polymerisation of three co-monomers: methyl methacrylate, a PEG ester of methacrylic acid and a polysiloxane-bearing ester of methacrylic acid. The polysiloxane ester was synthesised in a sequence of reactions (Figure 16) and, instead of the typical dimethylsiloxane repeat unit, was composed of methyl (3,3,3-trifluoropropyl)siloxane repeat units. The amphiphilic nature, resulting from the two types of grafts, was found to bestow excellent protein adsorption resistance to the material, making it a promising candidate for biomedical applications.

### 4.4. Other Systems Bearing Siloxane Moieties

**Figure 17:**
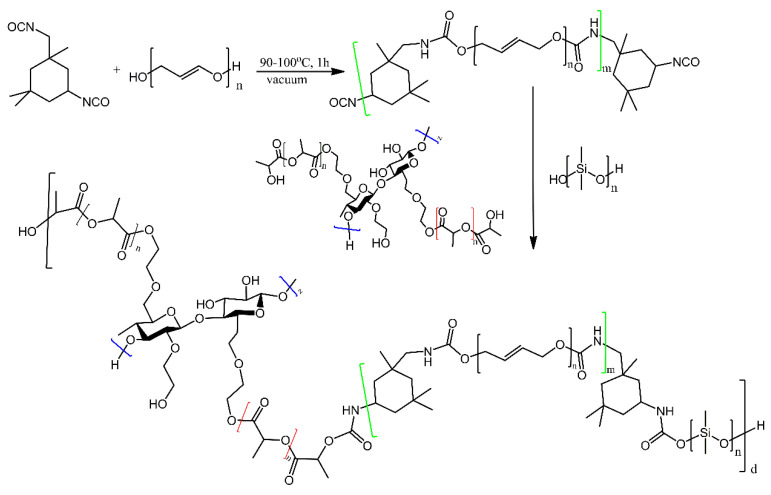
Schematic route of copolymerysation of PDMS based polyurethane. The different types of polymeric fragments are highlighted in colour for clarity. Based on Ref. [41].

Polyurethane prepolymers were produced using isophorone diisocyanate and hydroxyl-terminated polybutadien [41]. These prepolymers were then treated with either only hydroxyl-terminated PDMS or with hydroxyl-terminated PDMS and hydroxyethylcellulose modified with poly (lactic acid) grafts, yielding PDMS-bearing polyurethanes (Figure 17). The inclusion of the grafted hydroxyethylcellulose was found to improve both antibacterial properties and biocompatibility of the materials in regards to those lacking the modified hydroxyethylcellulose segments.

**Figure 18:**
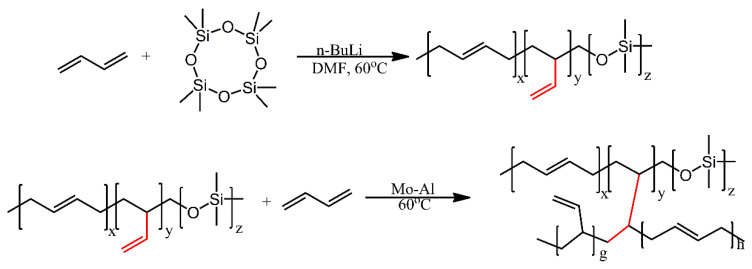
Schematic illustration for the preparation of 1,2-PB-g-Si. The vinyl group is highlighted in red for emphasis on its transformation. Based on Ref. [7].

A polybutadiene rubber, for application as a silica-filled elastomer, was produced by grafting a block copolymer of butadiene and dimethylsiloxane on polybutadiene [7]. The block copolymer was produced by sequential anionic polymerisation of butadiene and octamethyltetrasiloxane. The polymerisation reaction was not specific and produced both 1,2-linkage and 1,4-linkage between butadiene repeat units. Due to the presence of 1,2-linkages, the block copolymer was equipped with vinyl functionalities, which were utilised as active sites for grafting (Figure 18). The graft copolymer was produced by polymerising butadiene along with the produced block copolymer, in the presence of a standard molybdenum-based catalyst. The resultant graft copolymer was vulcanised in the presence of silica and the resultant rubber showed lower Mooney viscosity, as well as a more uniform silica distribution in comparison with standard polybutadiene rubbers.

**Figure 19:**
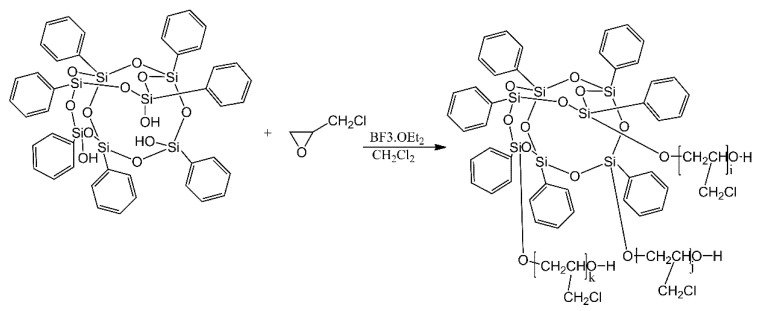
Schematic route of POS- polyepichlorohydrin. Based on Ref. [11].

POSS derivatives, equipped with polyepichlorohydrin grafts were synthesised as new materials for improving the damping properties of polyurethane-based composites [11]. First, a heptaphenyl-substituted polyhedral oligomeric silsesquioxane (POSS) was treated with epichlorohydrin in the presence of the adduct of boron trifluoride and diethyl ether (Figure 19). A series of graft copolymers were thus produced, with a variety of average molecular weights, depending on the reagent ratios used for polymerisation. Polyurethanes were then produced, using mixtures containing polyepichlorohydrin, castor oil and polyarylpolymethylene isocyanate, as well as one of the resultant graft copolymers. The use of graft copolymers in polyurethanes resulted in an increase of the damping factor (tanδ) from 0.90 for POSS-lacking polyurethanes to 1.16, while resulting in a slightly lowered glass transition temperature.

A polyfluorene-based copolymer, containing hydroxyoctyl substituents, was first treated with an isocyanate-functionalised silane and then with one of two polyetheramine derivatives, in order to produce fluorene-bearing di- or triureasils, depending on the functionality of the employed polyetheramine derivative. These ureasils were designed in order to alter the molecular organisation of the conjugated polyfluorene chains and, consequently, their various optoelectronic properties. Although the photoluminescence properties of the polyfluorene copolymer were indeed found to change over the course of the abovementioned reactions, it should be noted that the photoluminescence intensity also decreased significantly and that the effect of this modification on the conductivity of the materials was not investigated [42].

The operation of dielectric elastomer actuators [43] relies on the coulombic attraction of the oppositely charged electrodes, located on opposite sides of the elastomer material. This attraction induces a stress on the polymer in the direction of its thickness and results in an expansion of the elastomer in a plane parallel to the planes of the electrodes. Due to their favourably high electrical permittivity, polysiloxanes and their derivatives have found applications in the manufacture of such devices.

## 5. Conclusions

Despite the many developments in polysiloxane chemistry and the evolution of various synthetic procedures, the traditional method of producing siloxane bonds—via the reaction of silanes with various types of hydroxyl groups—remains predominant among the reviewed works. This is particularly so for the purpose of surface functionalisation with a variety of moieties. Other synthetic approaches, such as ring opening polymerisation of cyclic oligosiloxanes, are becoming more frequently reported and, in the case of the reported syntheses of graft copolymers, with either siloxane grafts or main chains, more sophisticated synthetic pathways are finding application. Currently, we are yet to see a broader usage of those reactions (Figure 1), even though many of them could realistically supersede the traditional synthetic approaches, particularly where well-defined polysiloxane systems are desired. As such, we believe that exploring this matter presents a significant opportunity for the development of the entire field of polysiloxane materials.

Regarding the applications of the various polysiloxane materials, medical applications, such as for the manufacture of low-fouling and bacteria-resistant surfaces, are the most prominent, particularly so in light of the current global situation. Nevertheless, the more classical applications involving the inclusion of polysiloxane segments, particularly in graft copolymers, such as the modification of the hydrophobic/hydrophilic nature of other materials, remain well-represented. Among emerging and growing applications, the use of polysiloxanes in actuators is particularly worth mentioning, as it actively utilises the dielectric properties of polysiloxanes.

In terms of methodology, it is worth mentioning that, although the works presented herein typically have systems whose structure has been unequivocally established using multiple analytical methods (Table 1), this is not the case for all recently reported works. A variety of analytical methods are also used for the multi-faceted investigation of the properties of the multitude of the reported systems.

## Figures and Tables

**Table 1:** Characterisation methods.

Ref.	Structural	Properties Confirmed by*
[1]	IR, ^1^H NMR	CV, FS
[2]	ATR-FTIR, IRRAS, XPS	CAM
[3]	XPS	EIS
[4]	IR, XPS	SEM, UV-Vis, TG, DTG
[5]	IR, XPS	
[6]	^1^H, ^13^C-NMR, IR	SEM
[7]	IR, GPC-MALLS, EA	
[8]	^1^H NMR, XPS, GPC	CAM, AFM
[9]	VT-IR ^1^H ^13^C NMR	CD, SAXS, POM, DSC, DLS
[11]	IR, ^1^H ^13^C NMR, TEM, SEM	DMA
[12]	XPS	SEM, hydrophilicity by CA.
[13]	^1^H NMR, XPS, EI-MS	UV−Vis, CV
		
[15]	IR, XPS	TG-DSC, SEM
		
[16]	IR, XPS	SEM
[23]	IR, ^1^H NMR, EDX	DSC, SEM, TEM, TG, DTG, Dielectric Conductivity, N2-Sorption Measurements
[24]	^1^H, ^13^C, ^29^Si NMR	
[25]	IR	DSC, SEM
[26]	XPS, GPC	AFM
[27]	^1^H, ^13^C, ^29^Si NMR, SEC	CAM, TGA, DSC, DLS
[28]	SEC, ^1^H, ^13^C, ^29^Si NMR	
[29]	^1^H NMR, IR, RM, SEC	DSC
[30]	IR, GPC	DSC
[31]	IR, ^1^H, ^29^Si NMR,	
[32]	^1^H NMR, SEC	
[33]	^1^H NMR, SEC	DSC
[34]	IR, SEC	
[35]	IR,	DSC, TGA, CA, UV-Vis (Transparency)
[36]	^1^H, ^13^C, ^29^Si NMR, IR GPC HRMS	
[37]	IR, ^1^H NMR, SEC	CAT
[38]	IR	SEM, EDS, CAT
[39]	^1^H, ^13^C, ^29^Si NMR, SEC	DSC, AFM
[40]	IR, ^1^H NMR	Particle size
[41]	IR and solid-state ^1^H NMR	
[42]	^1^H NMR and solid-state ^13^C, ^29^Si NMR	TGA, PXRD, UV/Vis, PL

* Abbreviations are explained in the relevant abbreviations section.

## Abbreviations

^1^H NMR	proton nuclear magnetic resonance
^13^C NMR	carbon 13 nuclear magnetic resonance
^29^Si NMR	siloxane 29 nuclear magnetic resonance
AFM	atomic force microscopy
AIBN	Azobisisobutyronitrile
ATR-FTIR	attenuated total reflection Fourier transform infrared spectroscopy
BTA	benzene-1,3,5-tricarboxamide
CA	dynamic contact angle measurements
CAM	contact angle measurement
CAT	contact adhesion testing
CV	cyclic voltamperometry
DFLS	dynamic light scattering
DFT	density functional theory
DMA	dynamic mechanical analysis
DSC	differential scanning calorimetry
DTG	differential thermal gravimetry
EA	elementary analysis
EDX	energy-dispersive X-ray spectroscopy
EI MS	electrospray ionization mass spectroscopy
EIS	electrochemical impedance spectroscopy
FS	fluorescence spectroscopy
GPC	gel permeation chromatography
HRMS	high-resolution mass spectra
IR	infrared spectroscopy
IR RAS	infrared reflection-absorption spectroscopy
ITO	indium-tin oxide
MHS	methylhydrosiloxane
MW	molecular weight
PDI	polydispersity index
PDMS	poly(dimethylsiloxane)
PDMS-co-MHS	poly(dimethylsiloxane-co-methylhydrosiloxane)
PL	photoluminescence spectroscopy
POM	polarized optical microscopy
POSS	polyhedral oligomeric silsesquioxanes
PTFE	poly(tetrafluoroethylene)
PVAc	poly(vinyl acetate)
PXRD	powder X-ray Diffraction
RAFT	reversible addition-fragmentation chain transfer
RM	raman spectroscopy
SAXS	small angle X-ray scattering
SEC	size exclusion chromatography
SEM	scanning electron microscopy
TG	thermal gravimetry
UV-Vis	ultraviolet–visible spectroscopy
VT-IR	variable temperature infrared spectroscopy
XPS	X-ray Photoelectron Spectroscopy
XRD	X-ray Diffraction
